# Suppressive effects of *Momordica charantia* MAP30 on the senescence, proliferation and migration of bladder cancer cells mediated by CENPA

**DOI:** 10.1038/s41598-025-14977-y

**Published:** 2025-08-13

**Authors:** Kun Lu, Liu Chao, Jin Wang, Xiangyu Wang, Longjun Cai, Jianjun Zhang, Shaoqi Zhang

**Affiliations:** 1 Department of Oncology, the Affiliated Suqian Hospital of Xuzhou Medical University, Suqian, China; 2 Department of Urology, the Affiliated Suqian Hospital of Xuzhou Medical University, Suqian, China; 3 Suzhou Medical College of Soochow University, Suzhou, Jiangsu China

**Keywords:** *Momordica charantia*, MAP30, Cell senescence, CENPA, Traditional Chinese medicine, Cancer, Drug discovery

## Abstract

Bladder cancer (BC) is notably prevalent, particularly among men. Emerging evidence highlights that traditional Chinese medicine (TCM) has distinct anticancer properties. This study focuses on recombinant MAP30, a key active constituent of* Momordica charantia* extract, recognized for its antiviral, immunomodulatory, and antitumor effects. We discovered that MAP30 suppresses BC cell proliferation and induces apoptosis in a dose-responsive manner. It also hinders cell migration, as evidenced by Transwell and wound healing assays. In nude mice, MAP30 injections adjacent to tumors significantly curtail tumor growth. Molecular analyses after MAP30 exposure show elevated SA-β-Gal activity and increased expression of cell cycle inhibitors p21 and p16 in BC cells. Integrating TCGA BC data, MAP30 appears to correct dysregulated gene expression associated with the cell cycle, senescence, apoptosis, and key pathways such as FOXO, TNF, and MAPK. Proteomic analysis identifies CENPA as a central molecule inhibited by MAP30. Correlation studies reveal CENPA’s significant association with oncogenic and immune-modulatory gene expression and immune cell infiltration. CENPA knockdown diminishes BC cell growth and motility, while its overexpression in MAP30-treated cells reverses these effects, suggesting CENPA’s pivotal role in MAP30’s anticancer activity and its potential as a therapeutic target. In conclusion, recombinant MAP30 effectively hampers bladder cancer cell proliferation and migration via CENPA downregulation and induces apoptosis and senescence, offering therapeutic potential against bladder cancer.

**Supplementary Information:**

The online version contains supplementary material available at 10.1038/s41598-025-14977-y.

## Introduction

Bladder cancer (BC) is one of the most common cancers, ranking fourth among all cancer types in men and eleventh among women. BC is also the second most common malignant tumor of the urinary system in the world, with approximately 549,000 new cases and 200,000 deaths annually^1,2^. Despite many improvements in surgical and radiotherapeutic methods, as well as the widespread use of perioperative chemotherapy, the long-term survival rate of BC patients has not significantly improved for decades. At the same time, advanced molecular research and bioinformatics technologies based on big data have greatly increased our understanding of this disease^3^. It is believed that in the future, individualized treatment for tumor patients, combined with the improvement of surgical techniques and the improvement of new treatment methods, may bring better therapeutic effects for bladder cancer patients.

More and more evidence suggests that traditional Chinese medicine (TCM) has unique effects on cancer treatment^4^, but there is a lack of scientific research on its potential molecular mechanisms. Evidence-based medicine in traditional Chinese medicine indicates that traditional Chinese medicine targets cancer cells and treats the entire body^5^. Bioactive compounds from TCM can exert anti-tumor activity by targeting multiple levels and signaling pathways. Numerous evidence suggests that TCM has significant advantages over conventional therapies in terms of fewer side effects, lower toxicity, and lower economic burden^6,7^. In the past few decades, a series of studies have been conducted on traditional Chinese medicine, including chemistry, pharmacology, and clinical research, to better connect traditional Chinese medicine with Western medicine and gain credibility and reputation outside of China. Traditional Chinese medicine research, including anti- cancer research, follows two main paths: one based on purified natural products, and the other based on multi-component formulations, including single herbal preparations and prescription formulas^8^. The most famous case of traditional Chinese medicine in modern medicine may be the story of artemisinin, known for its outstanding anti-malaria effects and recognized by the Nobel Prize in 2015^9^. TCM is a potential treasure trove for drug discovery, especially for complex diseases such as cancer.

*Momordica charantia* is a common edible vegetable used in various ancient folk medical practices to treat some diseases^10^. Previous studies have shown that* Momordica charantia* extract (BME) serves as a natural AMPK activator to inhibit the growth of human cancer cells and induce cell apoptosis, without toxic effects in normal cells^11^. MAP30 is one of the most biologically active components in* Momordica charantia* extract, which has anti-human immunodeficiency virus (HIV), immune regulation, antiviral, and anti-tumor effects^12^. MAP30 has been reported to inhibit the proliferation and migration of various types of tumor cells, including ovarian cancer^13^, glioma^14^, colorectal cancer^15^ and liver cancer^16^. Here, we constructed a recombinant MAP30 protein expression plasmid and purified it through fermentation engineering technology to obtain the MAP30 recombinant protein. The purpose of this study was to explore the effect of recombinant MAP30 on the proliferation and invasion of bladder cancer cells and to analyze its anti-tumor mechanism through high-throughput data and bioinformatics analysis.

## Materials and methods

### Synthesis and identification of MAP30

The balsam pear leaves used in this study were provided by the Suzhou Institute of Nano-Tech and Nano-Bionics, Chinese Academy of Sciences. The genomic DNA of balsam pear leaves was extracted using the cetyltrimethylammonium bromide (CTAB) method using fresh emerald green and glossy thick balsam pear leaves. The MAP30 coding sequence was amplified by polymerase chain reaction (PCR) using upstream primer 5’- ATGAATTCATGGATGTAACTCGATTTGTC-3’ and downstream primer 5’- ATCTCGAGGTCAATTCACAAGATCC-3’. After restriction endonuclease digestion, the recombinant expression vector pET28a-MAP30 was constructed by cloning into the pET28a plasmid. Transforming the recombinant expression plasmid into the expression strain BL21, and the expression of recombinant MAP30 was induced using IPTG (Isopropyl β-D-Thiogalactoside). The total protein was obtained by lysing the bacteria, and the MAP30 recombinant protein expressed in BL21 was further purified by NTA-Ni^2+^resin, and the protein was identified by sodium dodecyl sulfate-polyacrylamide gel electrophoresis (SDS-PAGE).

### Cell culture

Human BC cell line 5637 and T24, as well as bladder epithelial cells SV-SUC-1 were purchased from the American Type Culture Collection (ATCC). All cells were cultured in DMEM (Thermo Fisher Scientific, Inc.) with 10% FBS (Thermo Fischer Scientific, Inc.) at 37 °C in the presence of 5% CO2.

### MAP30 dose-effect relationship curve

Gradient dilution of MAP30 was performed using DMEM medium containing 10% FBS to achieve final concentrations of 1.25, 2.50, 5.00, 10.00, 20.00, 40.00, and 60.00 µg/ml, respectively. 5637 and T24 cells were seeded into 96 well plates, with 8000 cells per well and 3 replicates per concentration. Discard the old culture medium after 24 h and add 200 µl prepared MAP30 working solution to each well separately and incubate under standard cultivation conditions for 48 h. Use the CCK-8 reagent kit to refer to the instructions of the reagent kit to detect the cell viability of each well and draw a dose-response curve.

### EdU cell proliferation assay

First, plant the cells in a 6-well plate with 1 × 10⁴ cells per well. Place appropriately sized cell slides into a six-well plate, digest the MAP30-treated cells with trypsin to prepare a single-cell suspension, and add them to the six-well plate. Incubate in a cell incubator for 24 h. Prepare EdU working solution according to the instructions of the reagent kit, add cells and incubate for 2 h before fixing and permeating the cells. Further incubation with fluorescent probes and nuclear staining was performed on the cells, followed by observation and photography under a fluorescence microscope.

### β-gal fluorescence imaging

Cell aging detection reagent SPiDER-β Gal was performed on cells β- Gal fluorescence staining, the brief steps are as follows: plant the cells in a 6-well plate with 1 × 10⁴ cells per well, after washing the cells with wash buffer, SPiDER- β Gal staining solution was added. The plate was incubated in dark conditions for 15 min and the cells were twice with PBS, followed by observation and photography under a fluorescence microscope.

### Western blotting

Total proteins were extracted from cells using RIPA lysing buffer (Beyotime Institute of Biotechnology, China) and the concentration of protein was determined using BCA assay (Beyotime Institute of Biotechnology, China). A total of 30 µg total protein was loaded and separated by SDS-PAGE and was then transferred onto a PVDF membrane (MilliporeSigma, Billerica, MA). Following blocked with 5% BSA, the membrane was incubated at 4 °C overnight with primary antibodies of Ki67 (dilution, 1:1,000), CDH1 (E-cadherin; dilution, 1:1,000), CDH2 (N-cadherin; dilution, 1:1,000) and ACTB (internal reference for cytoplasmic extracts; dilution, 1:1,000). After washed with TBST buffer, the membranes were then incubated with HRP-labeled secondary antibodies. The bands were visualized using the ECL substrate and images were collected using the GeneTools GBox (Syngene, Frederick, MD, USA) system. The intensity of each band was quantified using ImageJ software (NIH).

### Cell apoptosis analysis

First, plant the cells in a 6-well plate with 1 × 10⁴ cells per well. Digest each group of cells with trypsin digestion solution, terminate digestion with a complete culture medium, and centrifuge. Cell precipitation is resuspended with a complete culture medium to prepare a single-cell suspension. Centrifuge at 1000 rpm for 5 min and discard the supernatant. Wash the cell precipitation twice with pre-cooled PBS. Add Binding Buffer and FITC labeled Annexin V to the cell precipitates of each group, and incubate at room temperature in the dark for 30 min. Add 5 µl of 50 µg/ml PI, incubate in the dark for 5 min, then add 400 µl Binding Buffer. Perform flow cytometry analysis using BD FACS Canto II software.

### Wound healing assay

Single-cell suspension was prepared by digesting cells with a pancreatic enzyme digestion solution. Cells were inoculated into a six-well plate with 106 cells per well, and then cultured in a cell incubator for 24 h. When the cell grows to about 90%, use a 10 µl tip to create scratches at the bottom of the six-well plate. Add serum-free DMEM medium and culture in a cell incubator under conventional cell culture conditions. Perform image acquisition at 0, 24, 48, and 72 h. Analyze the scratch area using image J and calculate the scratch healing rate.

### Transwell migration assay

Cells were seeded in the upper chamber of the Transwell membrane (Corning, Inc., USA) with 1 ml FBS-free DMEM medium, and 2 ml complete medium was added to the lower chamber. Plant the cells in a 6-well plate with 1 × 10⁴ cells per well. After the cells were cultured at 37 °C for 48 h, the cells were fixed with 4% paraformaldehyde and stained with 0.5% crystal violet solution. Then, the cells on the upside of the Transwell membrane were wiped. Images of the migrated cells on the downside were taken under an inverted microscope and were then counted using NIH ImageJ software (Version 1.8.0).

### Soft agar colony formation assay

For soft agar colony formation (SACF) assay, cell suspension was mixed with 0.3% soft agar in DMEM containing 10% FBS and layered in triplicate onto 0.6% solidified agar in DMEM containing 10% FBS (1 × 10^3^ cells/well). After 14 days of culture, colonies containing 50 cells or more were counted under a microscope at magnification × 100 as previously described.

### Xenograft model

The nude mice used in this study were purchased from SPF (Suzhou) Biotechnology Co. Ltd. In total, 24 specific-pathogen-free grade male BALB/c-nude mice (age, 4 weeks) were housed in the barrier system of the animal center at Soochow University (Suzhou, China) and cared for following the National Institutes of Health Guide for the Care and Use of Laboratory Animals. Mice were housed and maintained in a specific pathogen-free (SPF) facility with 12 h light/dark cycles and free access to food and water. Animals did not experience undue suffering during the experiments. The protocol was approved by the Laboratory Animal Ethics Committee of the Experiment Animal Center of Soochow University (approval no. 202011A070). T24 cell suspensions were prepared via trypsinization and resuspended in PBS (5 × 10^7^ cells/ml), and 100 µl was injected subcutaneously into the right armpit of nude mice. On the day after injecting the cells, the control group was paratumor injected with PBS, while the experimental groups were injected with 10 µg/ml and 30 µg/ml MAP30, separately. All mice were euthanized at 21 days following implantation and the tumors were dissected. Tumor size was measured twice a week, and the maximum xenograft tumor size at the endpoint was less than 15 mm. Tissues were separated into two sections, whereby one was fixed with 4% paraformaldehyde for immunohistochemistry (IHC) and the other was stored at -80 °C for RT-qPCR or western blotting analysis. After the experiment was over, all nude mice were humanely euthanized by cervical dislocation, performed by trained personnel in accordance with institutional animal care guidelines and approved protocols.

### Immunohistochemistry

Tissue Sect. (0.3 μm) were deparaffinized and hydrated for staining. Following antigen retrieval, sections were blocked with 5% goat serum and 3% hydrogen peroxide. The sections were processed for IHC by incubating with specific primary antibodies, including CDH1 (1:100 dilution, ProteinTech Group, Inc., USA), CDH2 (1:100 dilution, ProteinTech Group, Inc., USA) and ki67 (1:100 dilution, ProteinTech Group, Inc., USA). Followed by incubating a goat anti-rabbit secondary antibody for 20 min and subsequently with streptavidin-HRP for 30 min at room temperature. After washing, the sections were developed using DAB and were counterstained with hematoxylin and dehydrated. Average optical density was quantified using the ImageJ plugin IHC profiler.

### Transcriptome profiling with microarrays

Use medium dose concentration, i.e. T24 cells were treated with MAP30 at a concentration of 10 µg/ml and collected at 48 h. We used the Trizol method to extract the total RNA from the sample and then used the NanoDrop 2000 equipment and Agilent Bioanalyzer 2100 instrument to perform quality inspection on the extracted total RNA. Only qualified samples obtained can enter the chip experiment. Chip-based high-throughput detection and data analysis were carried out at Jikai Biotech. The brief steps are as follows: quantitative analysis of total RNA samples was performed using Agilent 2100, and then the GeneChip 3’IVT Express kit was used to prepare aRNA (amplified RNA) according to the instructions. cDNA is then synthesized to obtain a double-stranded DNA template, which is further transcribed in vitro to obtain aRNA, which has been labeled by biotin. Purify the obtained aRNA and hybridize the fragmented RNA with the chip probe. After completion, wash and dye the chip, and finally collect images and raw data.

### Differential expression gene analysis

“limma” package of R software was used to conduct differential analysis on microarray data, and the screening criteria for differential genes were: |log2 (Foldchange)| > 1, adjust *P* value < 0.05. The high-throughput sequencing data of bladder cancer (BLCA) standardized by Transcript per Kilobase per Million mapped reads (TPM) were downloaded from The Cancer Genome Atlas (TCGA) database (https://portal.gdc.cancer.gov/) and used the “limma” package to perform gene differential expression analysis. The screening criteria for differentially expressed genes are: | log2 (Foldchange) | > 1, adjust *P* value < 0.05.

### Enrichment and protein-protein interaction analysis

Input differentially expressed genes into the metascape website (https://metascape.org/). After obtaining the enrichment analysis results, use the R program ggplot2 package for visualization. Through the String database (https://string-db.org/) analyze the protein-protein interaction network (PPI) of these differentially expressed genes, and use the Cytoscape plugin MCODE to screen out the top 4 clusters based on the degree level.

### Correlation analysis

To further explore the biological role and clinical significance of the CENPA, correlation analysis was performed between CENPA expression and oncogenes, Tumor Mutation Burden (TMB), Immune checkpoint gene expression, and immune cell infiltration. The oncogenes were extracted from ONGene database (http://www.ongene.bioinfo-minzhao.org)^17^. The 73 immunomodulatory genes (IMGs) were extracted from previous studies^18^. The correlation analysis was performed with the Spearman method based on “psych” package.

### Plasmid construction and transfection

The full length of CENPA was amplified using Pfu DNA Polymerase (Sangon Biotech Co., Ltd., China) and ligated into the pCDH overexpression vector (pCENPA). CENPA small hairpin (sh)RNAs were designed using the BLOCK-iT™ RNAi Designer. Subsequently, the shRNA pairs were annealed and ligated into the pGreen vector (shCENPA). The H1299 and S30 cells were transfected with pCENPA and shCENPA using Lipofectamine^®^ 6000 (Beyotime Institute of Biotechnology, China) according to the manufacturer’s protocol.

### Statistical analysis

GraphPad V8.3.0 software (GraphPad Software, LLC) was used for statistical analyses, and data were presented as the mean ± standard deviation. Student t-test and ANOVA (analysis of variance) were used to determine whether there was a statistically significant difference between the means of two or more groups, separately. For all statistical tests, *P* value < 0.05 was considered to indicate statistically significant.

### ARRIVE statement

This study was reported in accordance with the ARRIVE guidelines.

## Results

### MAP30 inhibits bladder cancer cell proliferation and promotes cell senescence

**Fig. 1:**
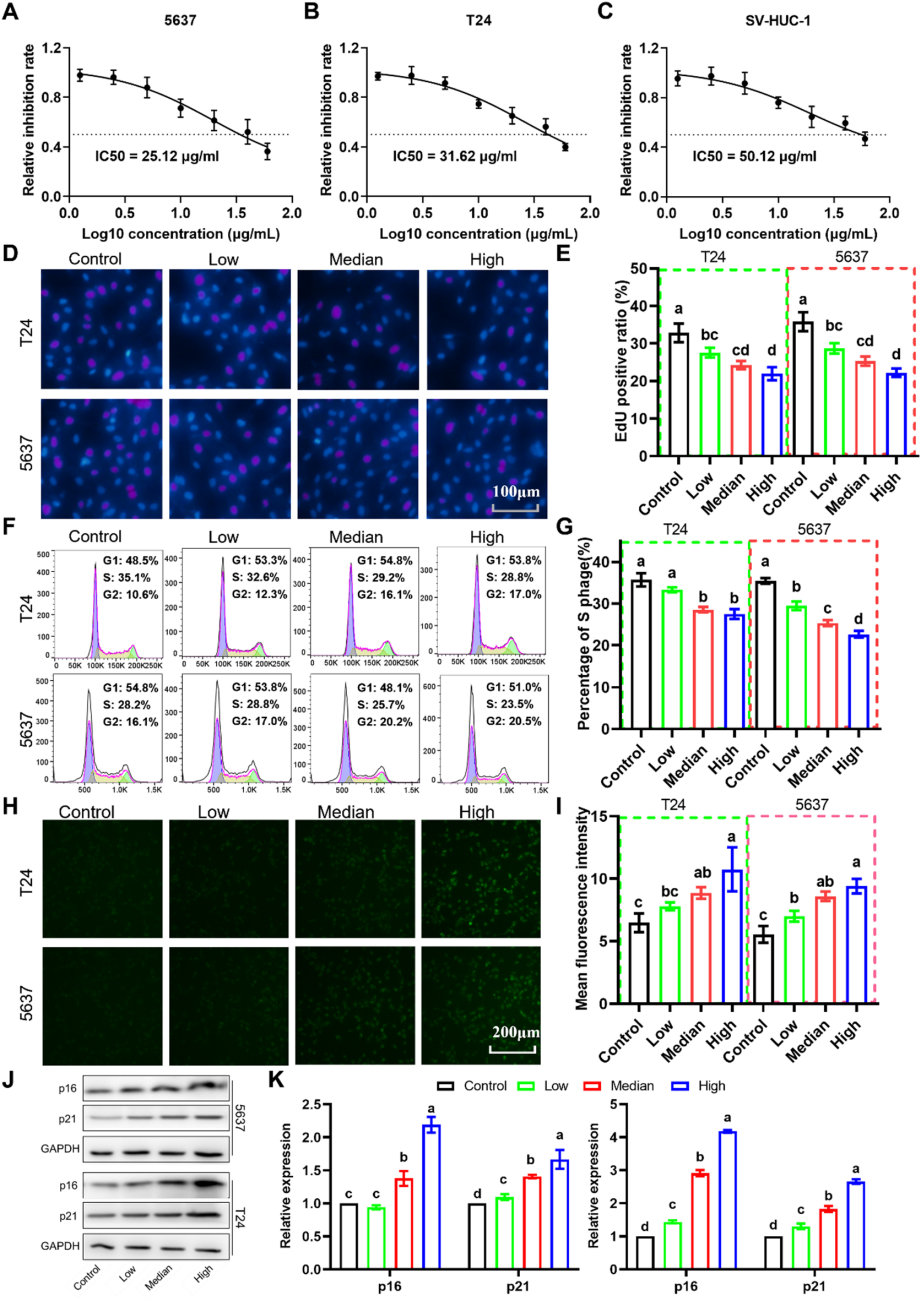
MAP30 treatment inhibits bladder cancer cell proliferation and promotes cell senescence. CCK-8 assay was used to measure the cell inhibition of MAP30 with different doses in 5637 (**A**), T24 (**B**), and SV-SUC-1(**C**). (**D**, **E**) Representative images of the results of the EdU assay and the quantitative results. (**F**, **G**) Representative images of the results of PI staining cell cycle assay and the quantitative results. (**H**,**I**) Representative images of the results of β-Gal immunofluorescence assay and the quantitative results. (**J**) Representative images of western blotting results of p16 and p21, and (**K**) the quantitative results. PI, propidium iodide. β-Gal, β-Galactosidase.

The MAP30 recombinant protein containing his tag at the C-terminus was expressed in BL21 (DE3) Escherichia coli and further purified to obtain MAP30 (Figure S1). As shown in Fig. 1, after treating three types of cells (5637, T24, and SV-SUC-1) with different concentrations of MAP30 (0、1.25、2.5、5、10、20、40、60 µg/ml), the cell proliferation showed a dose-dependent decrease, with IC50 of 25.12 µg/ml (5637 cells, Fig. 1A), 31.62 µg/ml (T24 cell, Fig. 1B) and 50.12 µg/ml (SV-SUC-1, Fig. 1C), respectively. Based on the dose-effect relationship curve, we selected three concentrations, viz. Low (2.5 µg/ml), Median (10 µg/ml), High (30 µg/ml) and solvent control (0 µg/ml) to perform subsequent toxicity testing and mechanism exploration.

Flow cytometry results showed that different concentrations of MAP30 treatment could significantly increase the apoptosis rate of 5637 and T24 bladder cancer cells in a dose-dependent manner (Figure S2). The EdU cell proliferation experiment showed that MAP30 treatment could reduce the EdU-positive cell ratio of both types (Fig. 1D, E). The cell cycle detection also showed that both median and high concentrations of MAP30 treatment significantly reduced the proportion of S-phase cells in a dose-dependent manner in cancer cells (Fig. 1F, G). β-Gal immunofluorescence assay was used to determine the level of cell senescence. The results showed that MAP30 treatment could promote cell senescence in a dose-dependent manner in both bladder cancer cells (Fig. 1H, I). At the molecular level, the expression of cell senescence-related proteins P21 and P16 was detected, and the results showed a significant increase in both after MAP30 treatment (Fig. 1F, G). These results indicate that MAP30 can inhibit the proliferation of bladder cancer cells and promote cell senescence.

### MAP30 inhibits bladder cancer cell migration

**Fig. 2:**
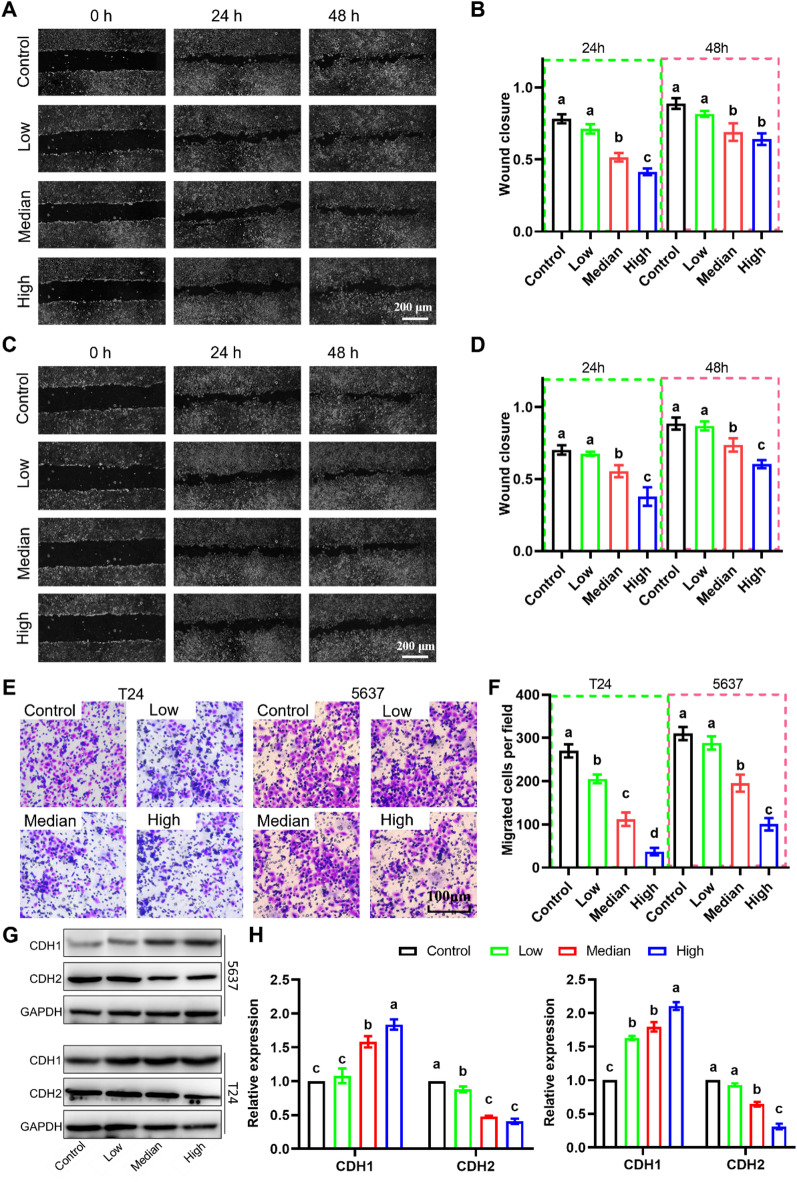
MAP30 treatment inhibits bladder cancer cell migration. (**A**,** B**) Representative images of the results of wound healing assay and the quantitative results in 5637 cells treated with MAP30. (**C**, **D**) Representative images of the results of wound healing assay and the quantitative results in T24 cells treated with MAP30. (**E**, **F**) Representative images of the results of the transwell migration assay and the quantitative results. (**G**) Representative images of western blotting results of CDH1 and CDH2, and (**H**) the quantitative results. CDH1, E-cadherin. CDH2, N-cadherin.

Next, we evaluated the effect of MAP30 on the migration ability of bladder cancer cells. The results of the cell scratch wound healing assay and transwell cell migration assay showed that medium and high doses of MAP30 treatment could significantly reduce the confluence rate of scratches of two bladder cancer cells, 5637 and T24 (Fig. 2A-D) and transwell cell migration rate (Fig. 2E, F) in a dose-dependent manner. At the molecular level, the expression of key genes for epithelial- mesenchymal transition, E-Cadherin (CDH1) and N-Cadherin (CDH2) was detected. In both 5637 and T24 cells, medium and high doses of MAP30 treatment significantly reduced the protein expression of CDH1, while the expression changes of CDH2 were opposite (Fig. 2G, H). These results indicate that MAP30 can inhibit the migration of bladder cancer cells.

### MAP30 inhibits tumorigenesis in vitro and in vivo

**Fig. 3:**
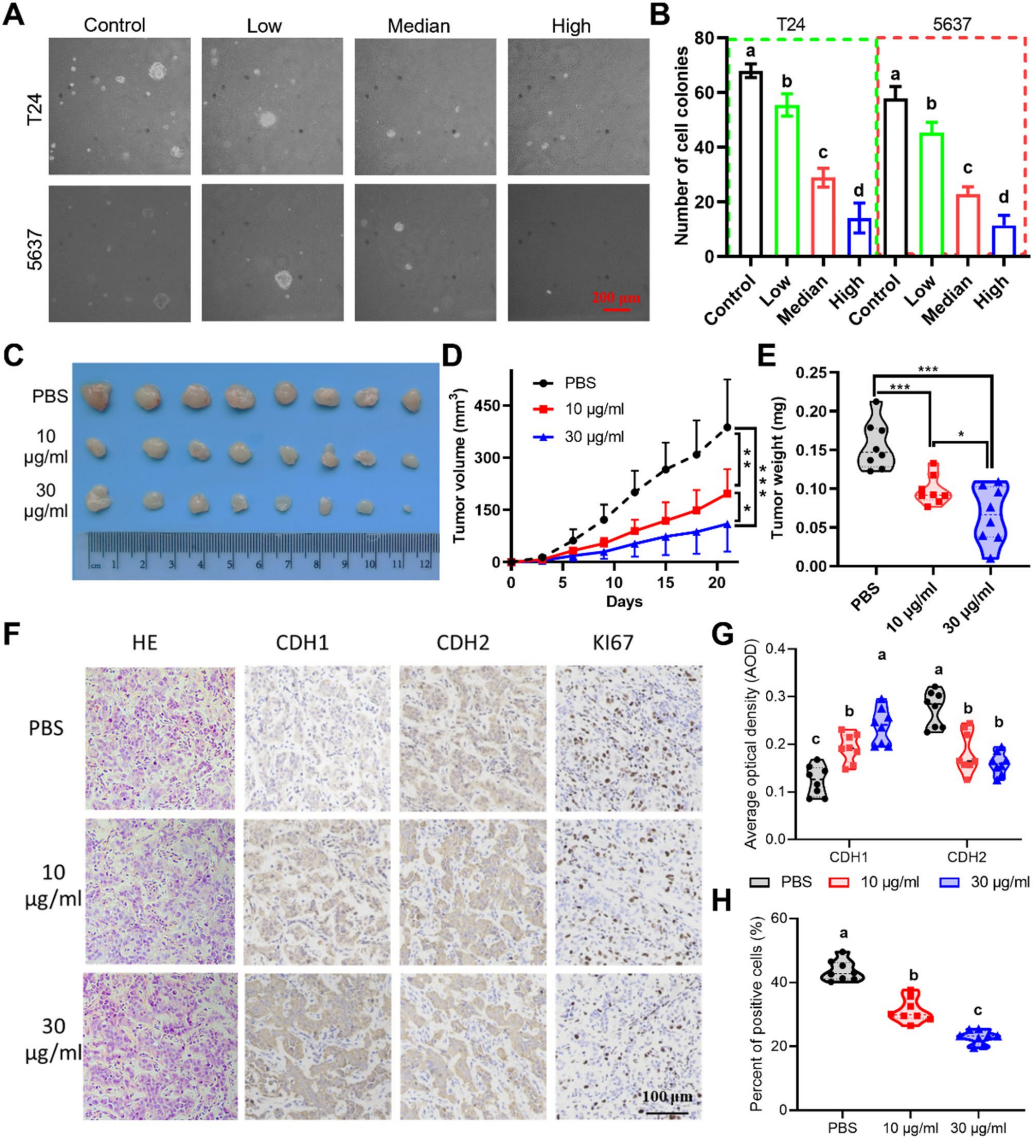
MAP30 inhibits tumorigenesis in vitro and in vivo. (**A**, **B**) Representative images of the results of soft agar clone formation assay and the quantitative results in 5637 and T24 cells treated with MAP30. (**C**) Images of subcutaneous tumors formed in nude mice subcutaneously injected with T24 cells and treated with different concentrations of MAP30. Statistical results of changes in (**D**) volume and (**E**) weight of subcutaneous tumors isolated from nude mice (*n* = 8). (**F**) Representative images of the results of HE and IHC staining of tumor sections. (**G**) Statistic results of the average optical density for CDH1 and CDH2. (**H**) Statistic results of the percent of Ki67 positive cells. CDH1, E-cadherin. CDH2, N-cadherin.

The soft agar clone formation assay showed that with the increase of MAP30 dose, the clone formation ratio of 5637 and T24 cells significantly decreased in a dose-dependent manner, and the size of the formed clones also decreased (Fig. 3A, B). To further verify the effect of MAP30 on the growth of bladder cancer cells in vivo, T24 cells were used to build a subcutaneous tumor model. The results showed that compared with the control group, the growth rate and mass of the tumor showed a significant decrease after paratumor injection of MAP30, and the inhibitory effect of 30 µg/ml is more significant (Fig. 3C, E). Further immunohistochemical analysis showed that compared to the PBS control group, paratumor injection of MAP30 with the concentration of 10 µg/ml and 30 µg/ml significantly upregulated the expression of CDH1, while CDH2 was significantly downregulated (Fig. 3F, G). For the proliferation marker protein KI67, its positive rate was also significantly downregulated (Fig. 3H).

### Effect of MAP30 treatment on the gene expression profile of bladder cancer cells

**Fig. 4:**
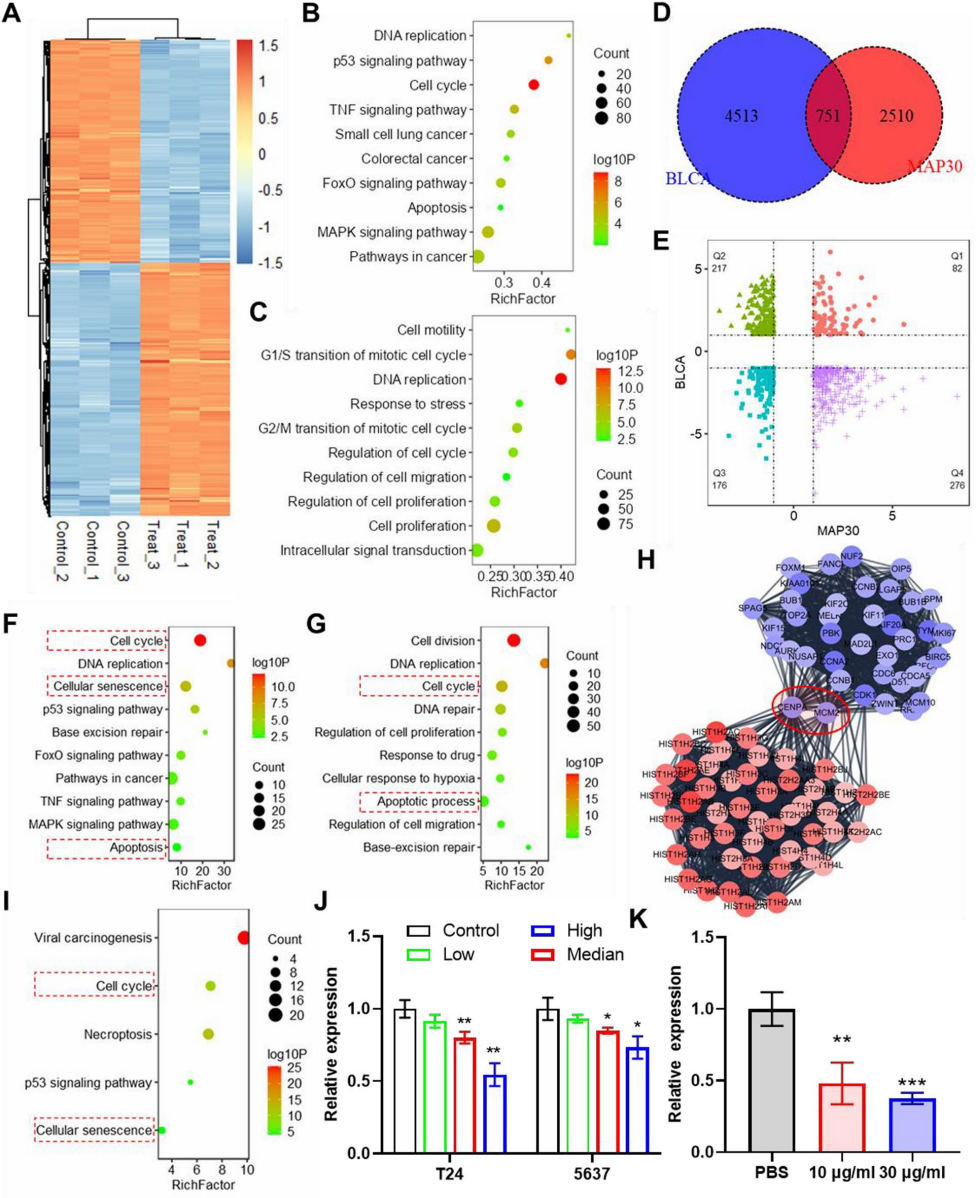
Effect of MAP30 treatment on the gene expression profile of bladder cancer cells. (**A**) Heatmap shows the DEGs in MAP30-treated T24 cells. Bubble plots show the enrichment analysis of (**B**) KEGG pathways and (**C**) biological processes for DEGs. (D)Venn diagram shows the intersection of DEGs in MAP30-treated cells and tumors. (**E**) The quadrant diagram shows the distribution of DEGs in MAP30 treatment and bladder cancer. Bubble plots show the enrichment analysis of (**F**) KEGG pathways and (**G**) biological processes for the DEGs fell in Q2 and Q4. (**H**) The key cluster of the PPI network was analyzed by the String database and MCODE plugin of Cytoscape software. (**I**) The bubble plot shows the enrichment analysis of KEGG pathways for the DEGs that fell in cluster 1. (**J**) CENPA relative expression in MAP30 treated bladder cancer cells. (**K**) CENPA relative expression in the tumors dissected from nude mice treated with MAP30.

Microarray analysis was performed on T24 cells treated with MAP30. After differential expression analysis, a total of 1573 downregulated mRNAs and 1735 upregulated mRNAs were identified after MAP30 treatment (Fig. 4A). Enrichment analysis shows that these genes are significantly enriched in multiple signaling pathways related to tumors, mainly including DNA replication, p53 signaling pathway, Cell cycle, FOXO signaling pathway MAPK signaling pathway and Apoptosis et al. (Fig. 4B). Similarly, these genes are also enriched in multiple important biological processes, mainly including Cell mobility, G1/S&G2/M transition of the mitotic cell cycle, DNA replication, Cell promotion, Regulation of cell cycle, and Regulation of cell migration (Fig. 4C). When taking the intersection with the DEGs in the TCGA BLCA dataset, a total of 751 genes were identified as the candidates that are regulated by MAP30 and contribute to BLCA (Fig. 4D). The quadrant diagram shows that there are 493 genes (Q2: 217, Q4: 276) with opposite changes in MAP30 treatment and bladder cancer (Fig. 4E). Further enrichment analysis revealed that these genes are related to several vital pathways and biological processes, including cell cycle, cellular senescence, apoptosis, and others (Fig. 4F, G). In addition, the String database is used to generate a protein-protein interaction (PPI) network for these genes, and further key modules in the PPI network are obtained through the MCODE plugin. The ranked first Cluster 1 is selected for subsequent analysis (Fig. 4H). Enrichment analysis suggested that the genes that fell in cluster 1 are enriched in the cell cycle, cellular senescence, and other pathways (Fig. 4I). CENPA, one of the key nodes in cluster 1, was considered the key gene MAP30 regulated, and it was found downregulated in MAP30-treated bladder cancer cells (Fig. 4J) and tumors (Fig. 4K). The above results revealed that MAP30 treatment can the gene expression profile in bladder cancer cells, and CENPA was identified as the key gene in the DEGs regulation network.

### CENPA has important biological and clinical significance in bladder cancer

**Fig. 5:**
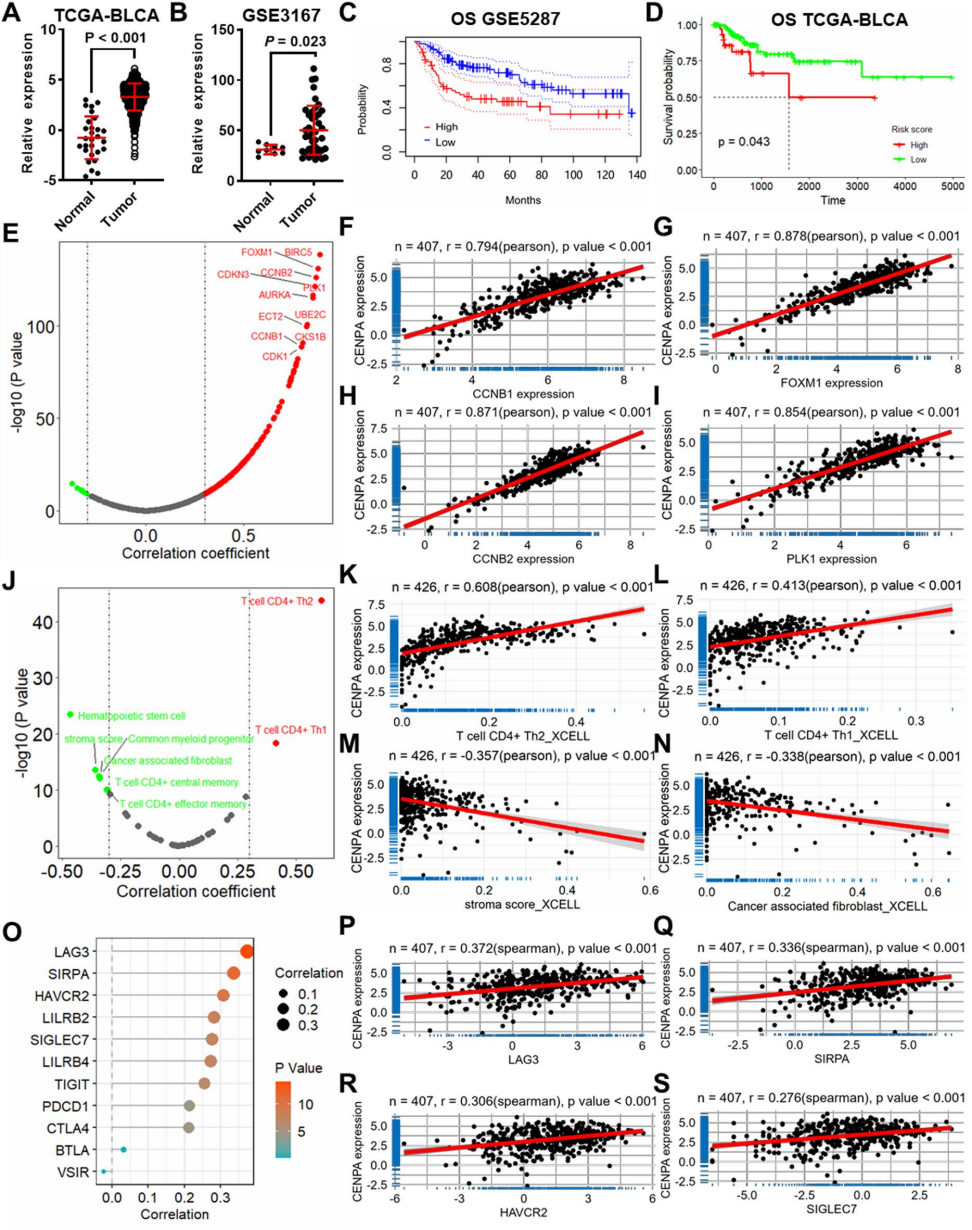
CENPA has important biological and clinical significance in bladder cancer. CENPA relative expression in bladder cancer based on TCGA-BLCA (**A**) and GSE3167 (**B**) datasets. Kaplan-Meier survival curves show the prognostic significance of CENPA for OS in GSE5287 (**C**) and TCGA-BLCA datasets (**D**). (**E**) The volcano plot shows the results of the correlation between CENPA and oncogenes. Scatter plots showing the correlation between CENPA and CCNB1 (**F**), FOXM1 (**G**), CCNB2 (**H**), and PLK1 (**I**).

As shown in Fig. 5A, B, the expression of CENPA was significantly lower in TCGA-BLCA and GSE3167 bladder cancer datasets. For the patient’s prognosis, the results show that CENPA contributes to the worse outcome of bladder cancer in both GSE5287 and TCGA-BLCA (Fig. 5C, D and Figure S3A-B) datasets. Also, CENPA was found high expressed in muscle-invasive bladder cancer based on the GSE13507 dataset (Figure S3C) and in high histologic-grade bladder cancer based on the TCGA-BLCA dataset (Figure S3D). To evaluate the ontogenetic role and clinical significance of CENPA, correlation analysis was performed between CENPA and the expression of oncogenes and immune cell infiltration based on the TCGA-BLCA dataset. CENPA was found significantly positively correlated with most oncogenes (Fig. 5E), mainly including CCNB1 (Fig. 5F), FOXM1 (Fig. 5G), CCNB2 (Fig. 5H) and PLK1 (Fig. 5I). In addition, CENPA is significantly correlated with the infiltration of several immune cells (Fig. 5J), mainly including Th2/1 CD4 + T cells (Fig. 5K, L), stroma-score (Fig. 5M) and cancer- associated fibroblast (Fig. 5N). The further correlation analysis between the expression of CENPA and immune checkpoints revealed that CENPA is significantly correlated with most checkpoints (Fig. 5O), including LAG3 (Fig. 5P), SIRPA (Fig. 5Q), HAVCR2 (Fig. 5R), SIGLEC7 (Fig. 5S). Moreover, a significant correlation was found between CENPA expression and tumor stemness (Figure S3E) and tumor mutational burden (Figure S3F) based on the TCGA-BLCA database. These results suggested that CENPA may serve as an oncogene in bladder cancer with important clinical significance.

### CENPA mediates the anti-tumor effect of MAP30

**Fig. 6:**
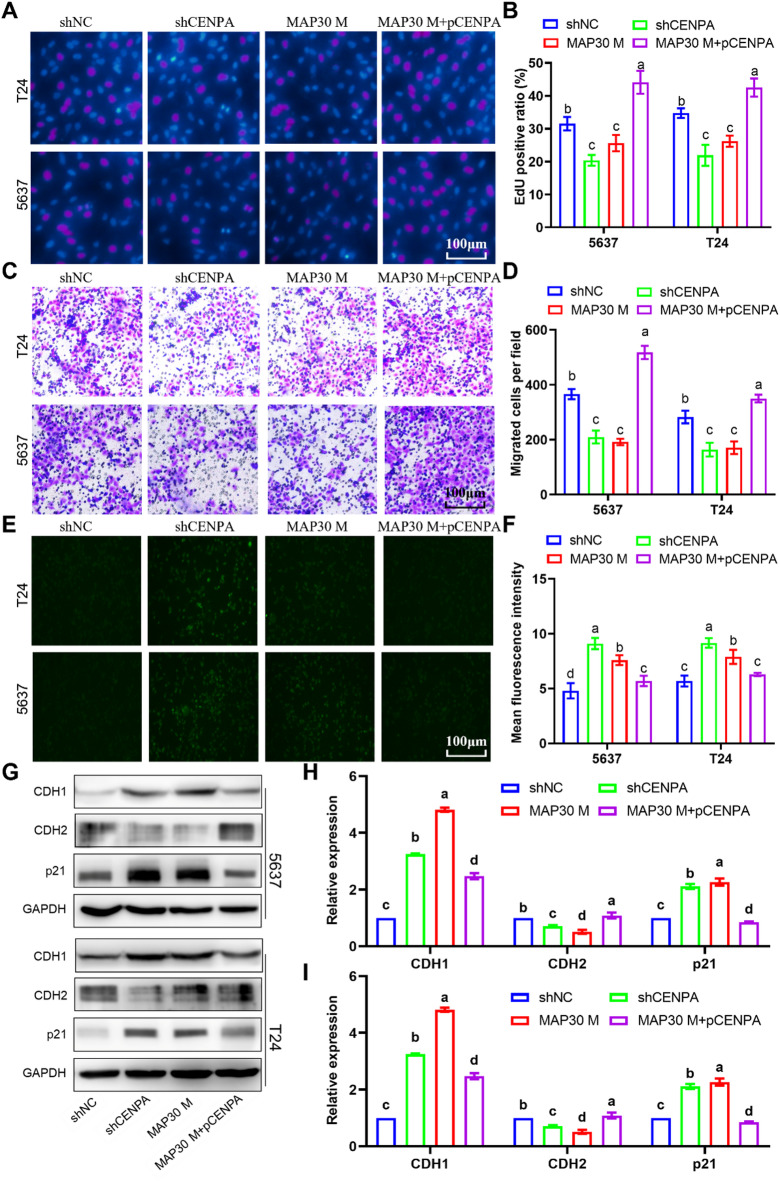
CENPA overexpression reverses the anti-tumor effects of MAP30. (**A**, **B**) Representative images of the results of the EdU assay and the quantitative results. (**C**,** D**) Representative images of the results of the transwell migration assay and the quantitative results. (**E**, **F**) Representative images of the results of β-Gal immunofluorescence assay and the quantitative results. (**G**) Representative images of western blotting results of CDH1, CDH2 and p21, and (**H**, **I**) the quantitative results. β- Gal, β-Galactosidase. CDH1, E-cadherin. CDH2, N-cadherin.

To predict the biological function of CENPA, GSEA enrichment analysis was performed based on the TCGA-BLCA dataset. The results revealed that CENPA was correlated with several cancer-related pathways and biological processes (Figure S5). As shown in Figure S6, CENPA overexpression and knockdown plasmids were constructed. The EdU cell proliferation assay showed that knockdown CENPA with shRNA transfection can significantly decrease the EdU-positive ratio of bladder cancer cells (Fig. 6A-B). When cells overexpressed with CENPA were treated with MAP30, the EdU positive ratio was significantly higher than that of cells treated with MAP30 alone, and significantly higher than that of cells overexpressed with CENPA (Fig. 6A- B). For cell migration, the transwell migration assay showed that CENPA knockdown significantly decreased the number of migrated cells (Fig. 6C, D). The EdU positive ratio was significantly higher in the CENPA overexpressed cells with the treatment with MAP30 than that of cells treated with MAP30 alone (Fig. 6C, D). When considering cell senescence, the β-gal staining showed that CENPA deprivation significantly promoted the activity of β-gal (Fig. 6E, F), while its activity was significantly inhibited in the CENPA overexpressed cells with the treatment with MAP30 than that of cells treated with MAP30 alone (Fig. 6E, F). The expression of CDH1, CDH2, and p21 was detected at the molecular level, and their changes were consistent with the changes at the cellular level. In the CENPA knockdown cells, the epithelial marker CDH1 and cell senescence marker p21 were significantly upregulated, while the mesenchymal marker CDH2 was downregulated, while CENPA overexpression can significantly inhibit the damage of MAP30 to bladder cancer cells (Fig. 6G-I). Also, CENPA was found to correlate with the sensitivity of several anti-tumor drugs (Figure S4A), including selumetinib, palbociclib, mim, tozasertib, bortezomib and other drugs (Figure S4B-G). These results revealed that CENPA knockdown can inhibit proliferation and migration, and CENPA overexpression can reverse the anti-tumor effects of MAP30.

## Discussion

Increasingly studies have found that TCM can target cancer cells and regulate the overall body, including blood, organs, and immune system^19^. The bioactive compounds in TCM can exert anticancer activity by regulating multiple targets in cancer cells^20^. Therefore, traditional Chinese medicine and its bioactive compounds may play an important role as ideal adjuncts in modern cancer treatment. Balsam pear is not only edible as food but also widely used for medicine in China and India. It can be used to improve digestion, treat diabetes and obesity, and is well-known for its “plant insulin” activity^21–23^. Previous research reports have found that* Momordica charantia* extract can inhibit ovarian cancer cells by activating the AMPK/mTOR signaling pathway^24^.* Momordica charantia* extract contains various bioactive compounds and has shown its anticancer effect in various human cancers^25–27^. For example, kuguacin J isolated from* Momordica charantia* extract reduced the resistance of ovarian cancer cells to paclitaxel therapy^25^, and RNase MC2 can induce apoptosis of human breast cancer cells^27^. A novel peptide BG-4 from* Momordica charantia* has also been reported to promote apoptosis in human colon cancer cells^26^.

MAP30 protein is one of the main components in* Momordica charantia* extract and has been reported to have anticancer effects in various types of tumors, including colorectal cancer^15^, ovarian cancer^13^, liver cancer^16^ and glioma^14^. Similarly, some studies have found that MAP30 drug can induce apoptosis of bladder cancer cells, and its mode is dose and time-dependent^28^. This study found that MAP30 can inhibit the proliferation of bladder cancer cells and promote the level of apoptosis in a dose-dependent manner. Transwell cell migration and wound healing assays also showed that MAP30 inhibited the migration ability of bladder cancer cells. It is well known that tumor metastasis is one of the main factors leading to poor prognosis in tumor patients. The inhibition of MAP30 on the migration ability of bladder cancer cells emphasizes its potentially important value in the treatment of bladder cancer. Further subcutaneous tumor formation models in nude mice indicate that paratumor injection of MAP30 can significantly inhibit tumor growth. These results are consistent with the previously reported anti-tumor effects of MAP30, but its mechanism is still unclear. Cellular senescence is a long-term and irreversible cell cycle arrest state triggered by in vitro and in vivo stress, characterized by specific secretory phenotypes, biomacromolecule damage, and metabolic changes^29^. Senescent cells also typically overexpress a hydrolytic enzyme in lysosomes β-galactosidase, known as “senescence-associated β-galactosidase (SA-β-Gal)”^30^. In this study, we found that after MAP30 treated bladder cancer cells, SA-β-Gal activity was enhanced, p21 and p16 expression were upregulated, and showed significant concentration dependence. This suggests that MAP30 may inhibit bladder cancer cells by promoting cell senescence.

As is well known, the specific process of the occurrence and development of diseases, including tumors, is very complex, especially reflected in the changes in complex regulatory pathways at the molecular level. Based on gene chip analysis, we identified 3308 differentially expressed genes in MAP30-treated cells. Based on the Integrative analysis of differentially expressed genes with TCGA bladder cancer data set, we found that MAP30 treatment saved some abnormally expressed genes in bladder cancer tissues. Further enrichment analysis revealed that these genes are associated with cell cycle, cell aging, and apoptosis, as well as FOXO, TNF, and MAPK signaling pathways. These results further emphasize the inhibitory effect of MAP30 on bladder cancer cells. Furthermore, based on PPI analysis, we identified the key molecule CENPA that MAP30 inhibits bladder cancer. CENPA is a critical determinant of chromosomal stability and the faithful execution of mitosis, with its principal function centered on the structural integrity and functionality of the centromere. As a histone H3 variant, CENPA exhibits distinct structural features and localization patterns, and is predominantly expressed in cells undergoing active mitotic division. In normal cells, suppression of CENPA expression has been shown to induce profound alterations in cell cycle progression, notably resulting in mitotic defects and arrest at the G1 or G2/M phases^31^. Furthermore, the regulatory influence of CENPA on the cell cycle appears to be closely linked to the p53 pathway. Inhibition of CENPA can lead to the activation of p53, thereby triggering G1/G2 phase arrest and promoting cellular senescence or apoptosis^32^. Analysis of paired normal tumor transcriptome from 101 datasets showed that overexpression of CENPA is a common feature in over 44% of human cancers^33^. CENPA has been implicated in the progression of various types of cancer through multiple regulatory mechanisms. For instance, CENPA can act as a transcriptional regulator to promote hepatocellular carcinoma progression by cooperating with YY1^34^. As a representative member of the centromere protein (CENP) family, CENPA overexpression has also been shown to drive renal cell carcinoma proliferation and metastasis by accelerating the cell cycle and activating the Wnt/β-catenin signaling pathway^35^. However, studies investigating the role of CENPA in the progression of bladder cancer remain limited. Based on the insights provided by previous findings, we sought to further explore the potential involvement of CENPA in bladder cancer development. Based on correlation analysis, we found that the expression of CENPA is significantly correlated with the expression of multiple oncogenes and immune checkpoint genes, as well as immune cell infiltration. Knockdown of CENPA in bladder cancer cells significantly decreased cell proliferation and migration. However, overexpression of CENPA in MAP30-treated cells could significantly rescue the down-regulation of cell proliferation and migration and the enhancement of cell senescence caused by MAP30 treatment, indicating that CENPA mediated the inhibitory effect of MAP30 treatment on bladder cancer cells.

## Conclusions

In conclusion,* momordica charantia* MAP30 can significantly inhibit the proliferation and migration of bladder cancer cells by downregulating the expression of CENPA and can promote cell apoptosis and cancer cellular senescence, thus playing a role in the treatment of bladder cancer.

This study is the first to reveal the therapeutic potential of recombinant MAP30 in bladder cancer and identifies CENPA as a novel downstream target mediating its antitumor effects. By integrating transcriptomic, proteomic, and functional analyses, we demonstrate that MAP30 suppresses bladder cancer progression through CENPA downregulation, linking cell cycle regulation, apoptosis, senescence, and immune modulation. These findings provide new mechanistic insights and support MAP30 as a promising candidate for bladder cancer therapy.

## Supplementary Information

Below is the link to the electronic supplementary material.

Supplementary Material 1

Supplementary Material 2

Supplementary Material 3

## Data Availability

Sequence data that support the findings of this study have been deposited in the GEO datasets with the primary accession code GSE3167 and GSE5287.
