# Supplementary material for: Suppressive effects of Momordica charantia MAP30 on the senescence, proliferation and migration of bladder cancer cells mediated by CENPA
Source: Sci Rep. 2025 Aug 13;15:29679. doi: 10.1038/s41598-025-14977-y (PMC12350783; doi:10.1038/s41598-025-14977-y)
Supplement: Supplementary file 1 — Supplementary Material 1 [file 41598_2025_14977_MOESM1_ESM.docx]

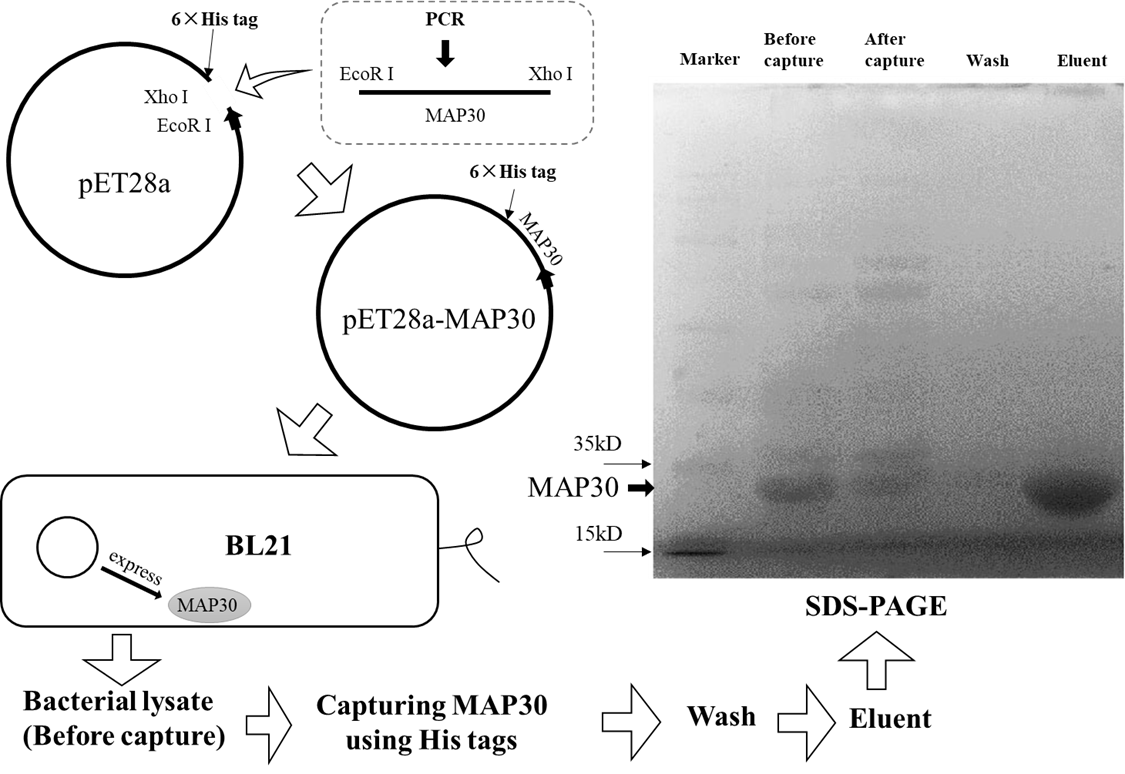


**Figure S1.** The process of constructing MAP30 recombinant protein


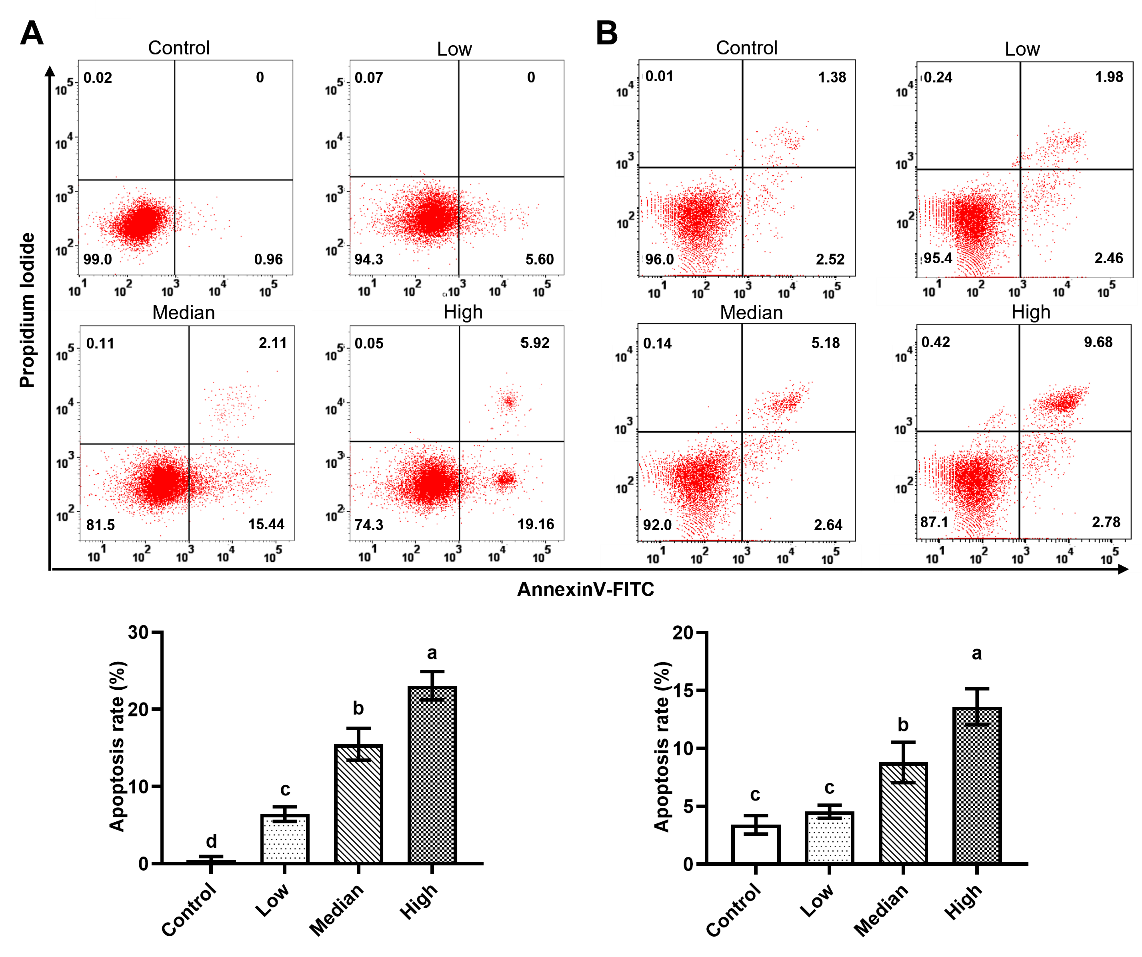


**Figure S2.** MAP30 treatment promotes bladder cancer cell apoptosis. Representative images of the results of cell apoptosis assay and the quantitative results in T24 (A) and 5637 (B).


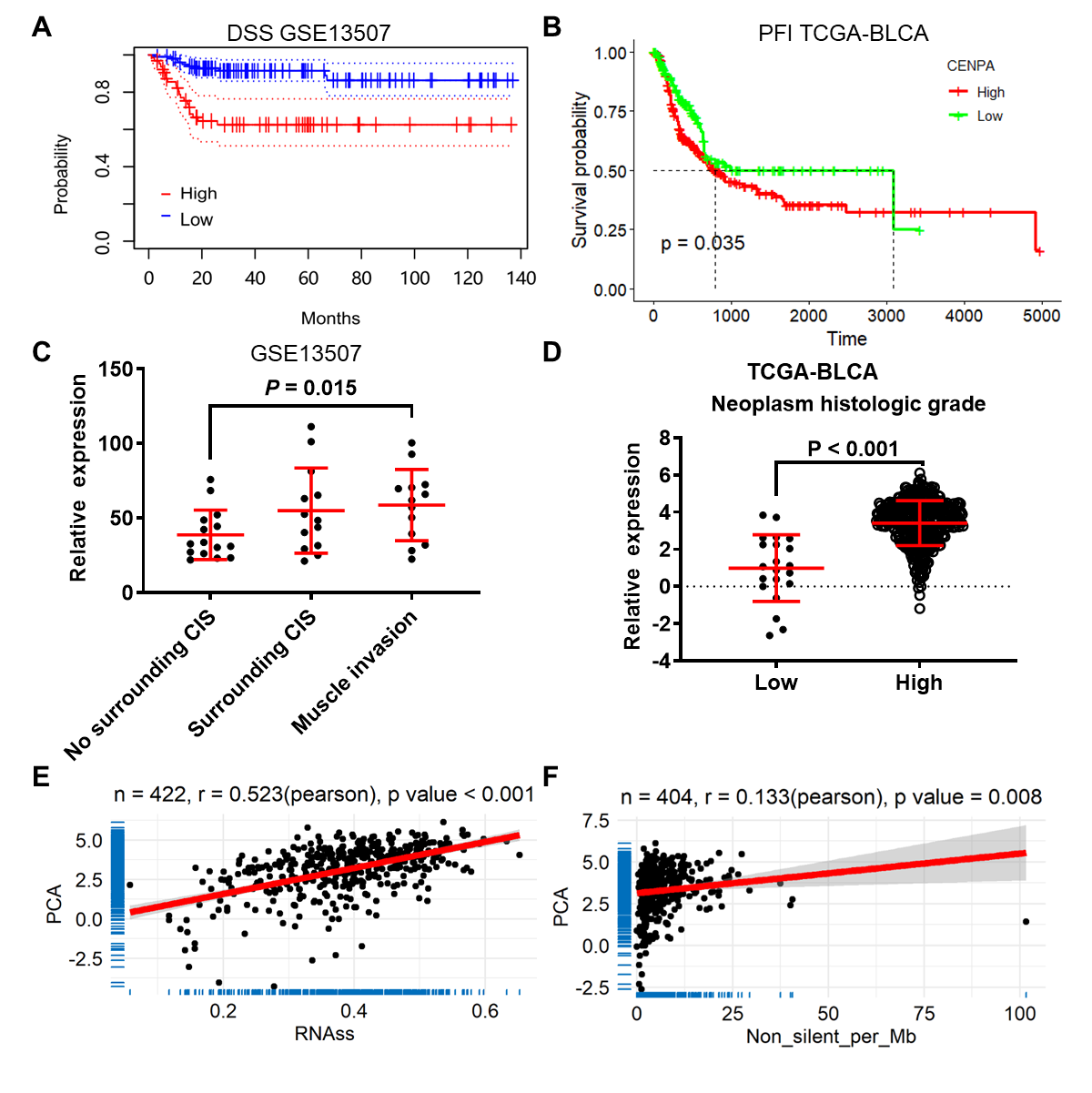


**Figure S3.** Bioinformatics analysis of CENPA in bladder cancer. (A) Disease-specific survival analysis of CENPA for bladder in GSE13507 dataset. (B) Relapse-free interval analysis of CENPA for bladder in TCGA-BLCA dataset. (C) Relative expression of CENPA in bladder cancer with different histologic grade. (D) Relative expression of CENPA in carcinoma in situ (CIS) lesions and muscle invasion bladder cancer. Scatter plots show the correlation analysis between CENPA and tumor stemness (E) and tumor mutational burden (F).


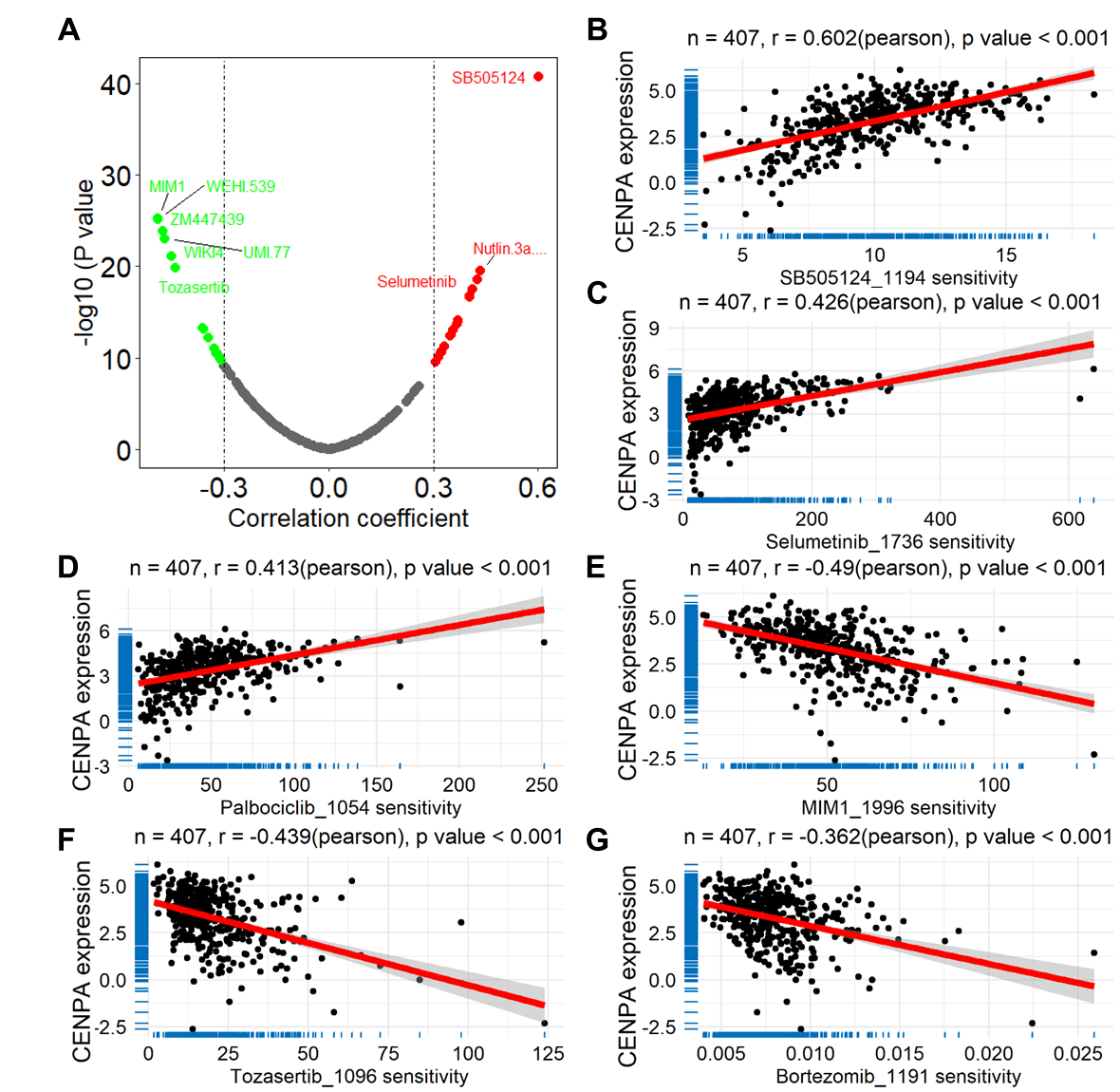


**Figure S4.** CENPA was correlated with the sensitivity of multiple anti-tumor drugs. (A) Volcano plot shows the results of the correlation between CENPA and the sensitivity of multiple anti-tumor drugs. Scatter plots showing the correlation between CENPA and the sensitivity of SB505124 (B), Selumetinib (C), Palbociclib (D), MIM1 (E), Tozasertib (F) and Bortezomib (G).


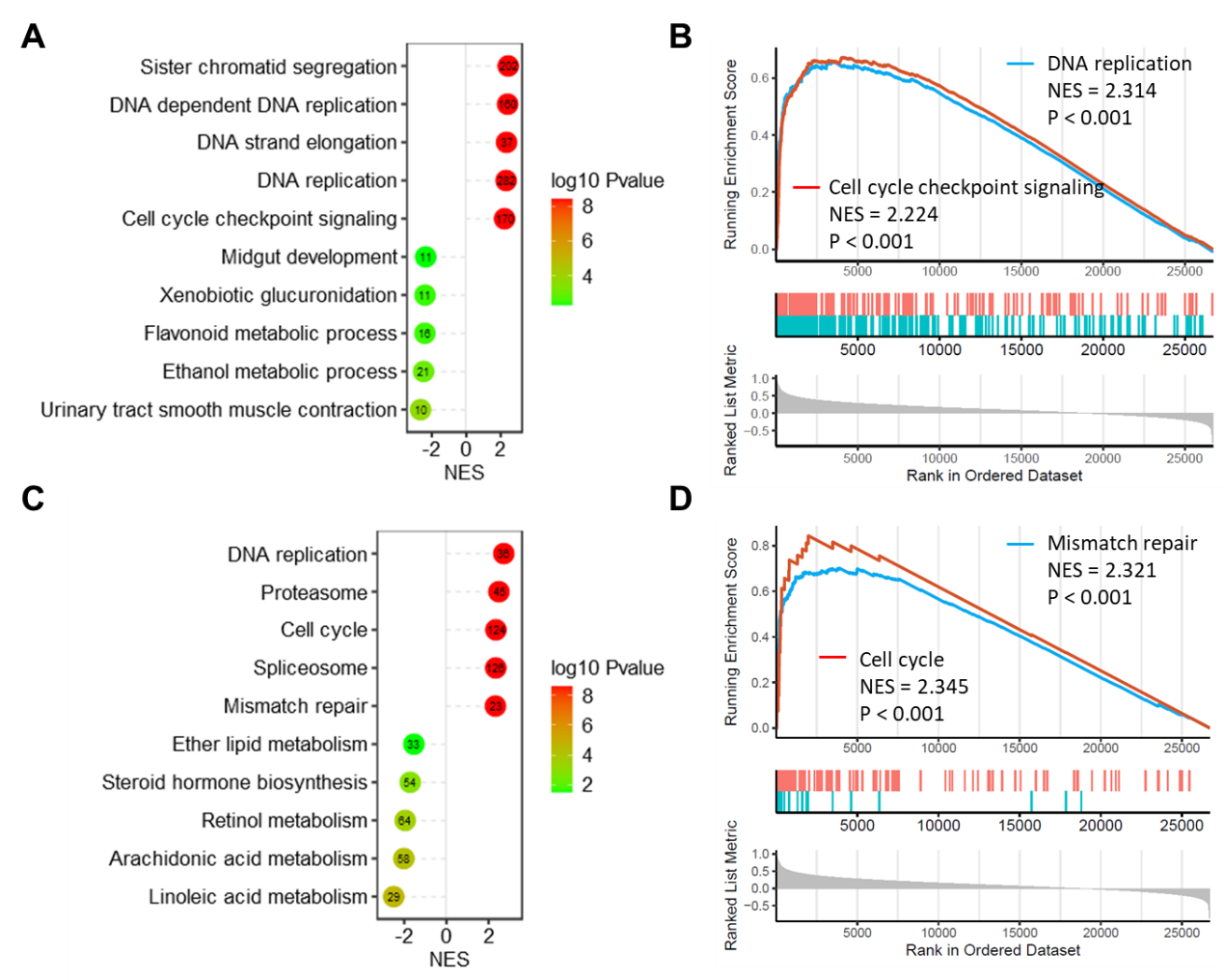


**Figure S5.** CENPA is related to many cancers associated biological processes and pathways. (A) Lollipop plot shows the GSEA result of CENPA for KEGG pathway. (B) GSEA plot shows CENPA was related with DNA replication and cell cycle checkpoint signaling. (C) Lollipop plot shows the GSEA result of CENPA for biological processes. (D) GSEA plot shows CENPA was related with mismatch repair and cell cycle.


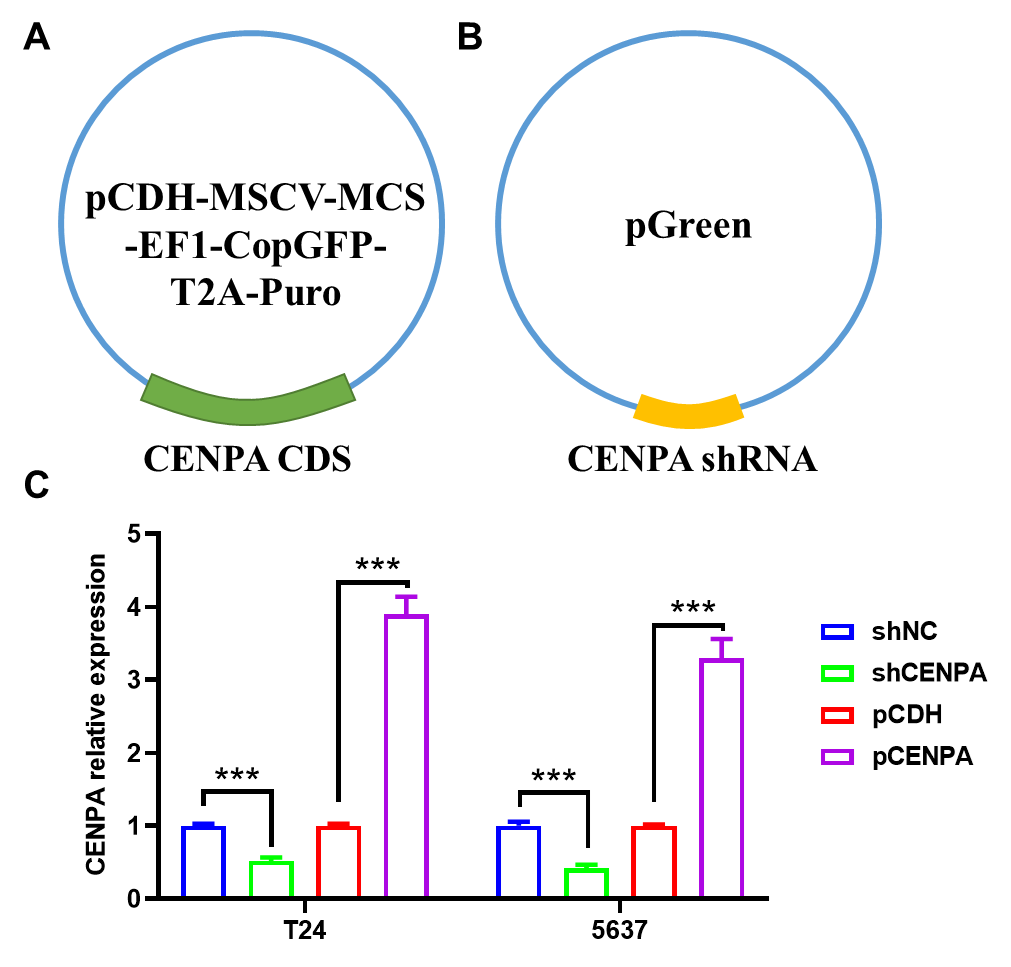


**Figure S6.** Construction of CENPA overexpression and knockdown plasmids. (A) Map of the construction of CENPA overexpression plasmid. (B) Map of the construction of CENPA knockdown plasmid. (C) Relative expression of CENPA in T24 and 5637 cells transfected with overexpression and knockdown plasmids.
