# Supplementary figures and images for: Suppressive effects of Momordica charantia MAP30 on the senescence, proliferation and migration of bladder cancer cells mediated by CENPA
Source: Sci Rep. 2025 Aug 13;15:29679. doi: 10.1038/s41598-025-14977-y (PMC12350783; doi:10.1038/s41598-025-14977-y)

CDH1\_1

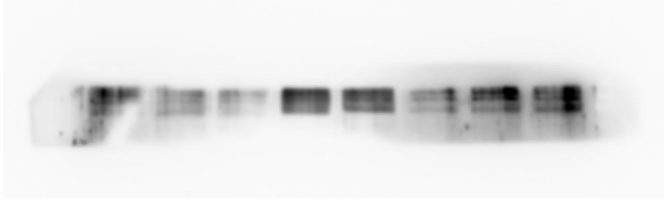

CDH1\_2

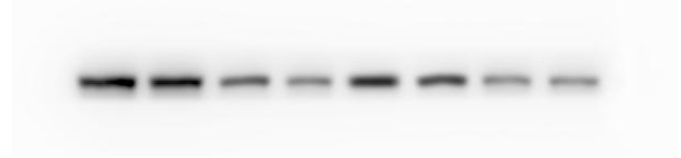

CDH1\_3

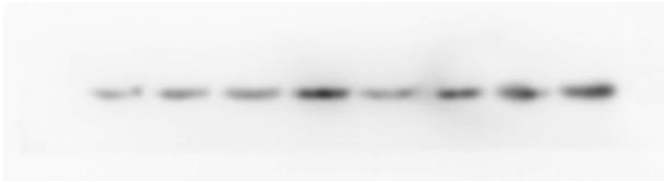

CDH2\_1

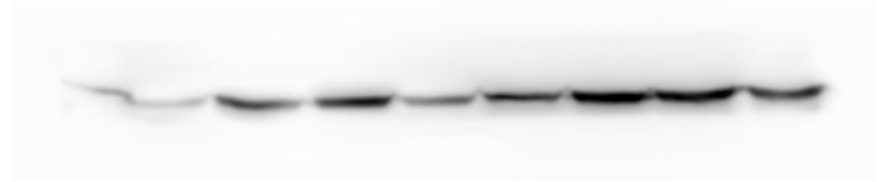

CDH2\_2

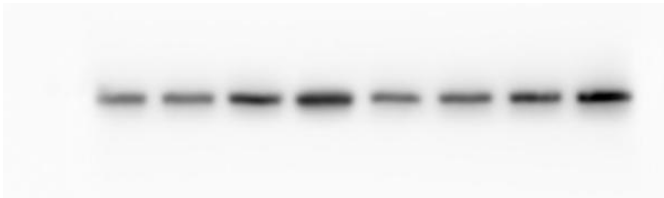

CDH2\_3

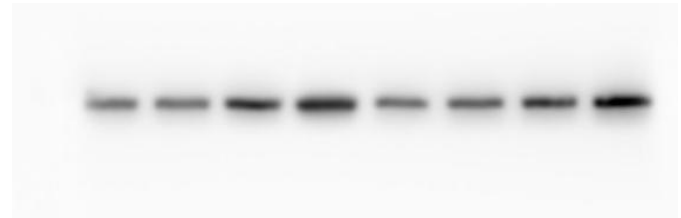

GAPDH\_1

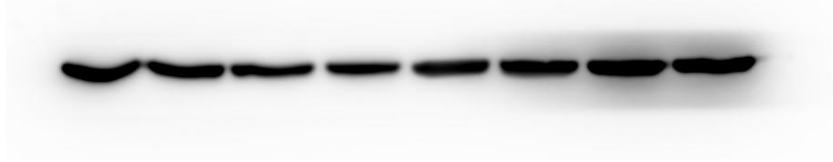

GAPDH\_2

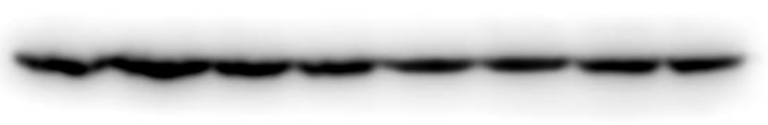

GAPDH\_3

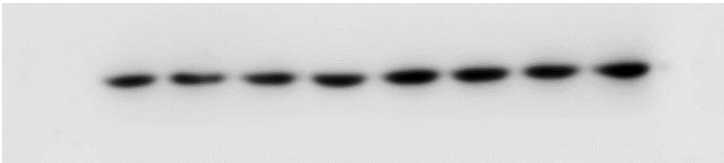

P21\_1

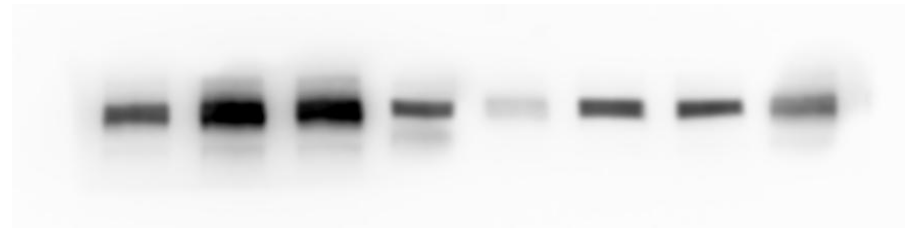

P21\_2

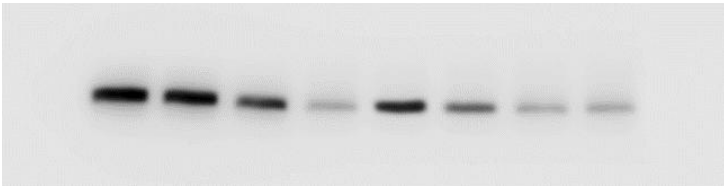

P21\_3

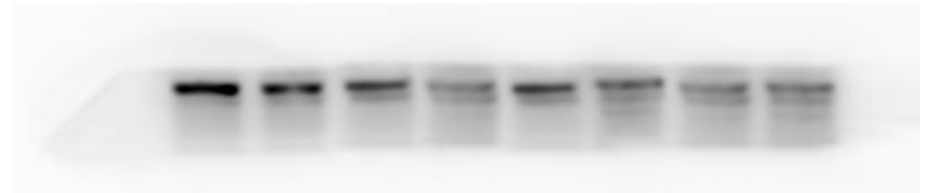

Supplement: Supplementary file 2 — Supplementary Material 2 [file 41598_2025_14977_MOESM2_ESM.pdf]

Figure 1J: 5637 cell

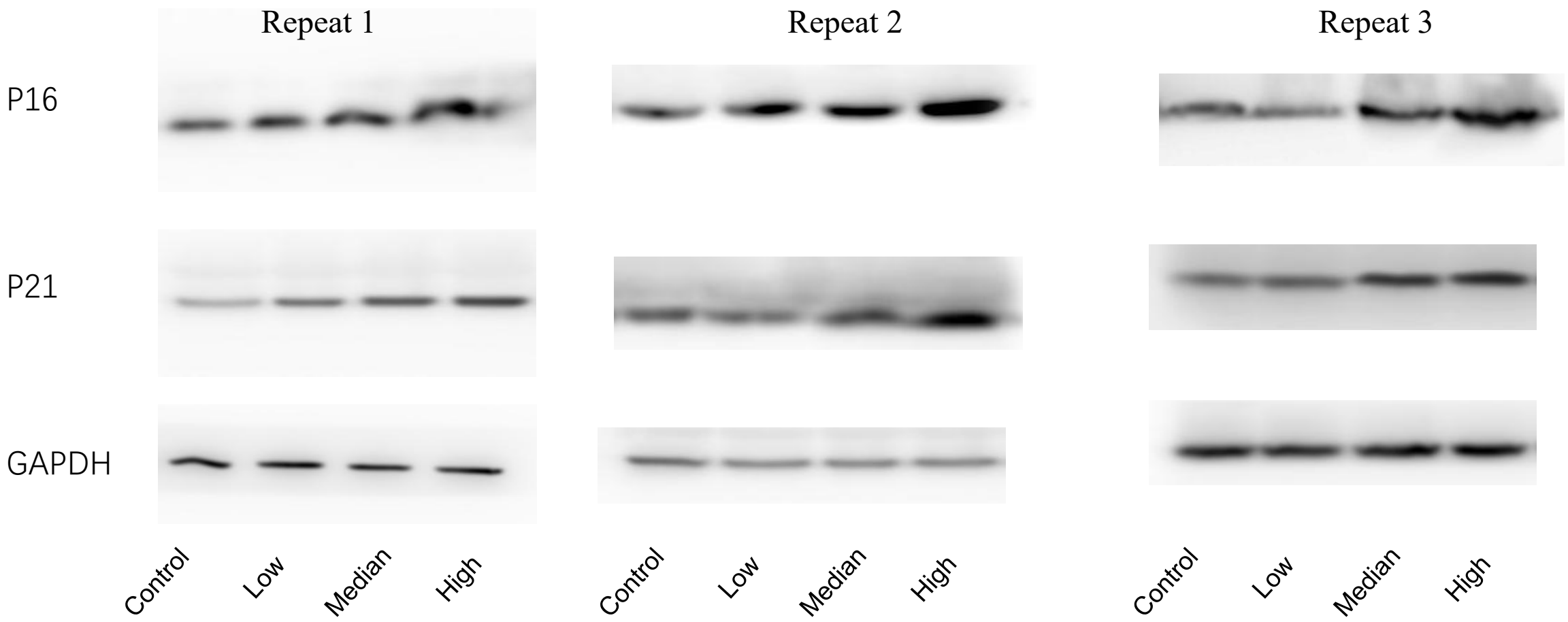

Figure 1J: T24 cell

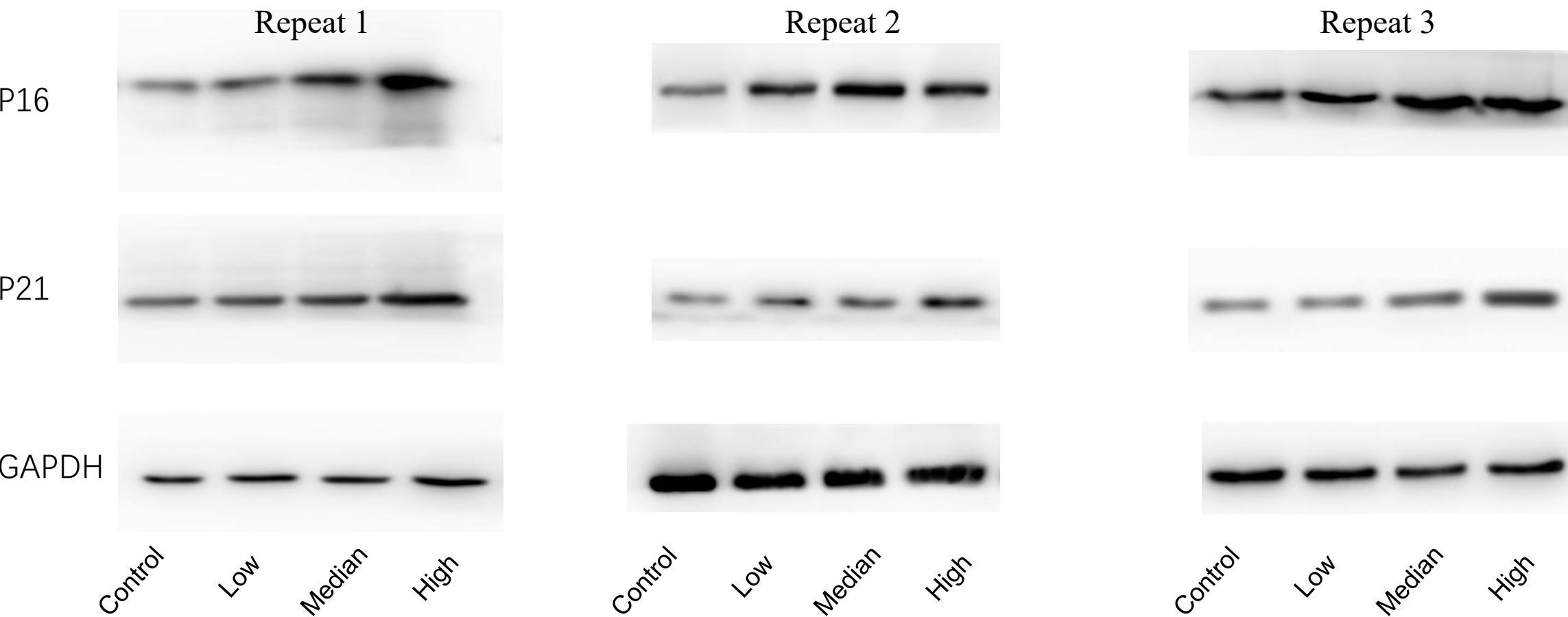

Figure 2G: 5637 cell

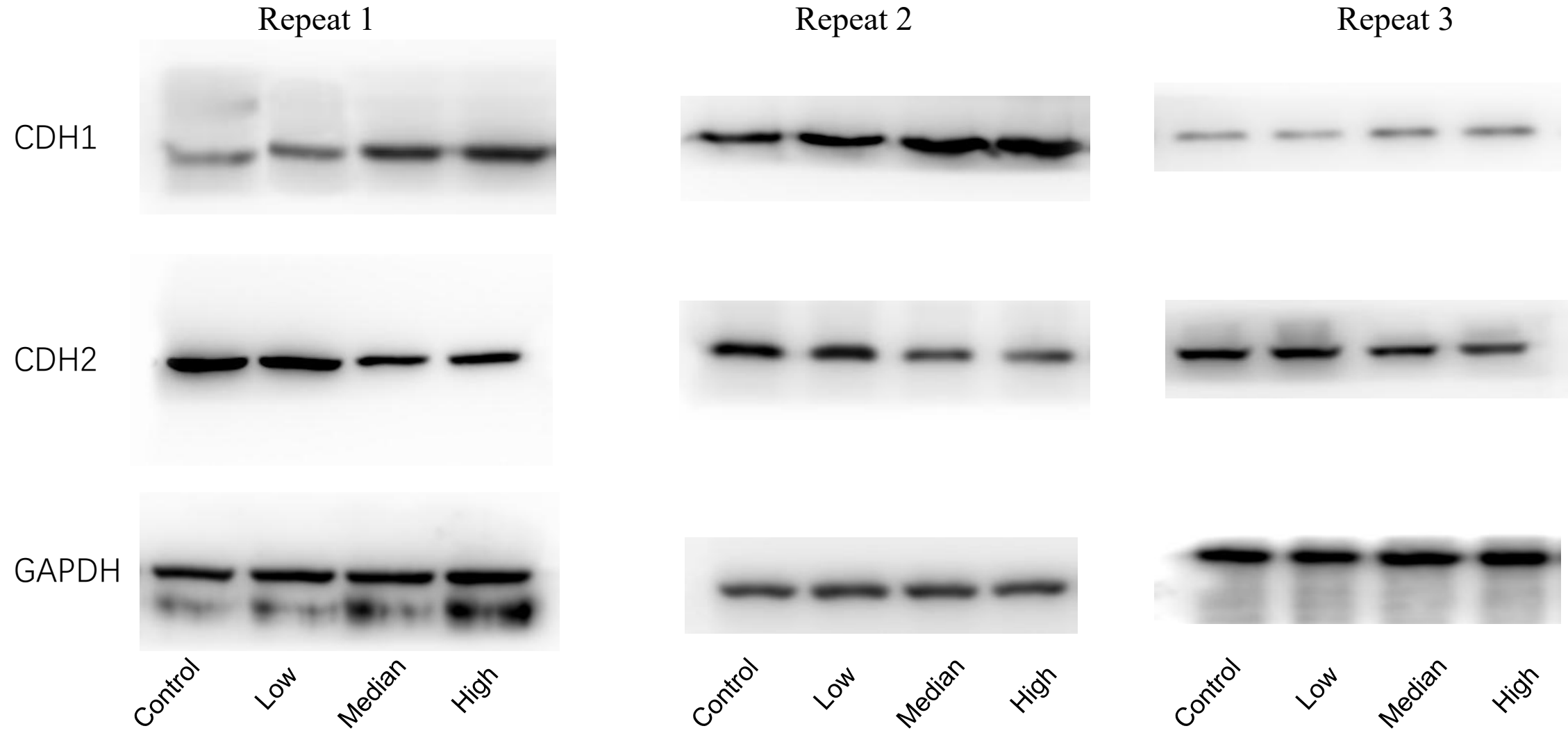

Figure 2G: T24 cell

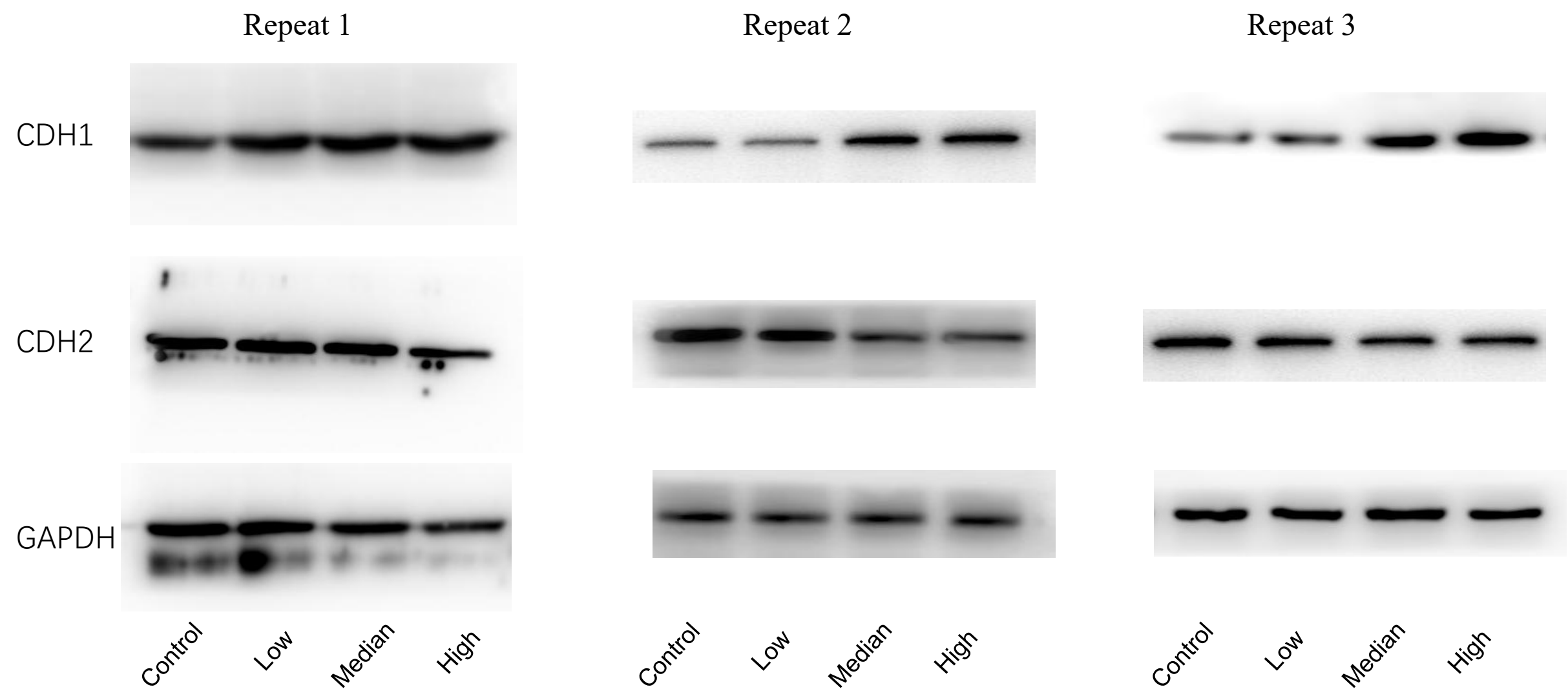

Figure 6G: 5637 cell

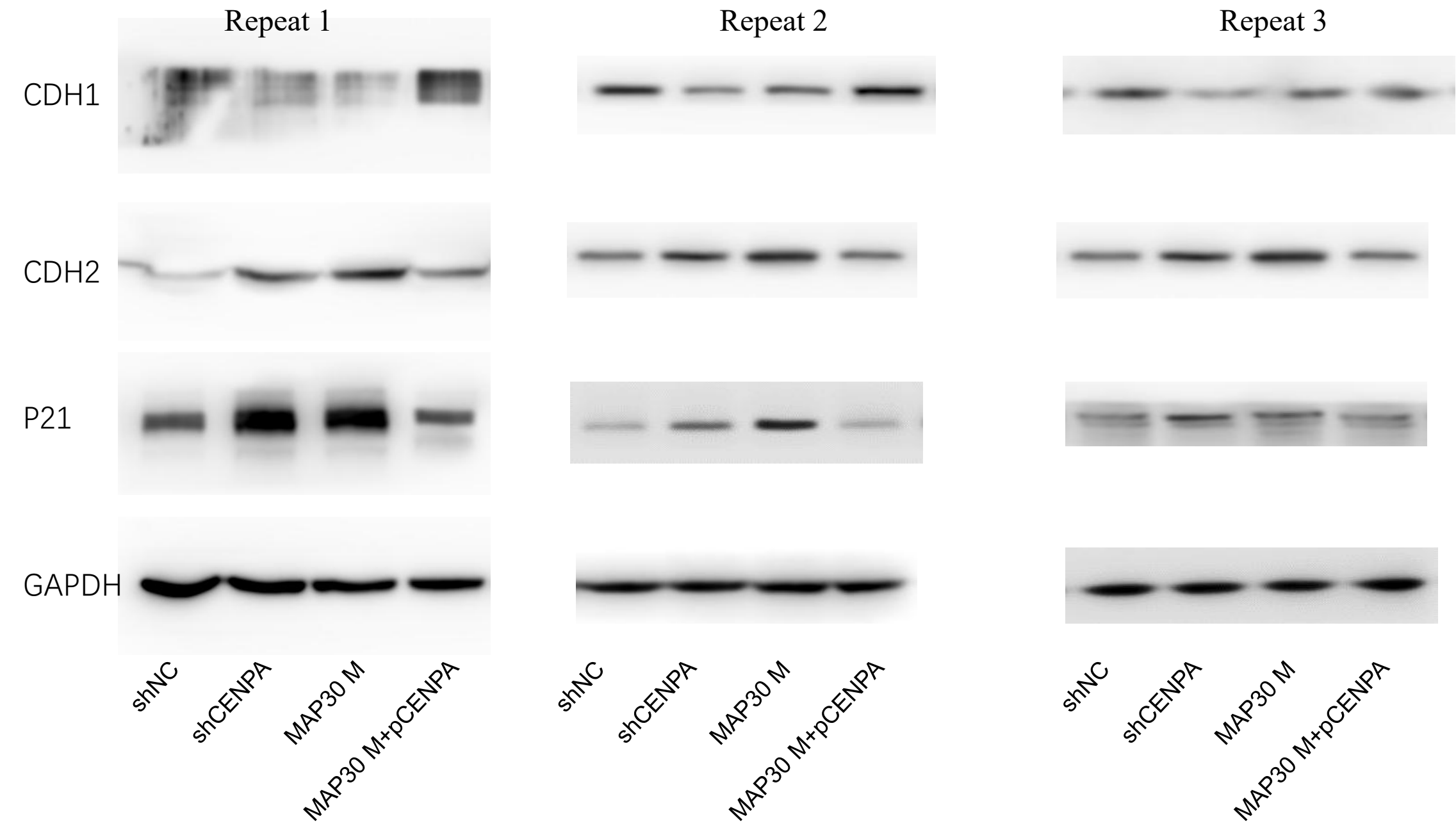

Figure 6G: T24 cell

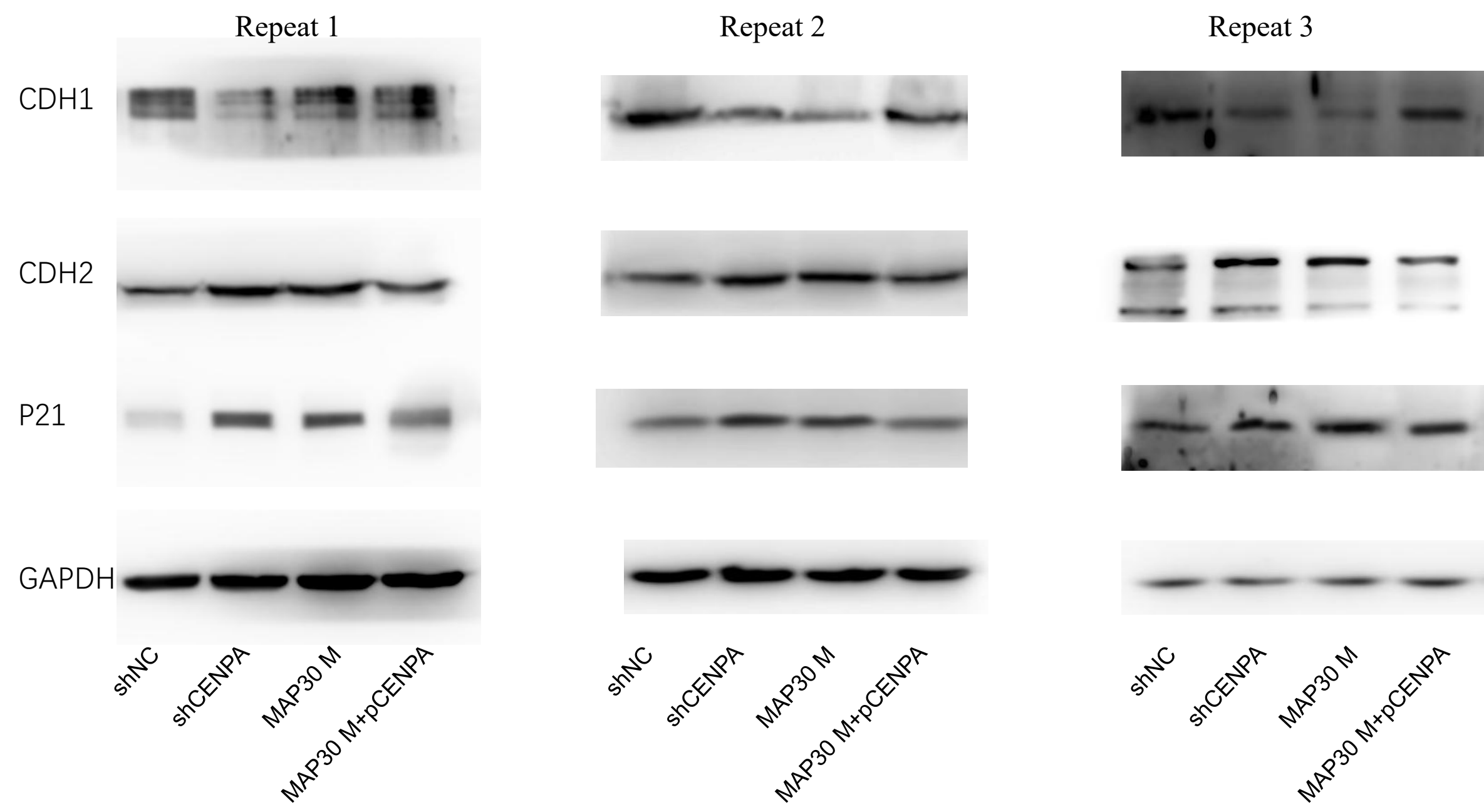

Supplement: Supplementary file 3 — Supplementary Material 3 [file 41598_2025_14977_MOESM3_ESM.pdf]
